# Are the concepts of emotion special? A comparison between basic-emotion, secondary-emotion, abstract, and concrete words

**DOI:** 10.3389/fpsyg.2022.915165

**Published:** 2022-09-13

**Authors:** Mauricio González-Arias, Daniela Aracena

**Affiliations:** Department of Psychology, Universidad de La Serena, La Serena, Chile

**Keywords:** emotional concepts, basic emotions, cause-and-effect attributes, concept attributes, perception of concression, abstract concepts

## Abstract

The study of emotional concepts stands at a very interesting intersection between the theoretical debate about the nature of emotions and the debate about the nature of processing concrete concepts and abstract concepts. On the one hand, it is debated whether it is possible to differentiate basic emotions from secondary emotions and, on the other hand, whether emotional concepts differ from abstract concepts. In this regard, the prototypical perceptual aspects are considered an important factor both for the differentiation between concrete and abstract concepts and for the differentiation between basic and secondary emotions (facial expressions). Thus, the objective has been to determine if (a) the presence or absence of a prototypical perceptual referent, and (b) the type of concept (referring to emotion and not referring to emotion), produce differences between concepts of basic emotions, secondary emotions and concepts not related to emotions, concrete and abstract, in the tasks of qualification of concreteness, imageability and availability of context and the task of the list of properties, that have been used in previous studies. A total of 86 university students from the suburbs of La Serena - Coquimbo (Chile), all native Spanish speakers, participated in the study. The results show that in the perception of concreteness and in the total of enumerated properties, emotional concepts presented similar results to abstract concepts not related to emotion and there was no difference between basic and secondary emotion concepts. In imageability and context availability, emotional concepts were perceived as different from and more concrete than abstract concepts. In addition, the cause-effect type attributes allowed to clearly differentiate emotional concepts from those not related to emotion and to differentiate between basic and secondary emotion concepts. These types of attributes appear almost exclusively in emotional concepts and are more frequent in basic emotions. These results are partially consistent with the predictions of Neurocultural and Conceptual Act theories about emotions.

## Introduction

The study of the concepts that refer to emotions is located at an interesting intersection between the theoretical discussion about the existence of basic emotions and the debate about whether emotion-related concepts differ from abstract concepts. Therefore, the concepts related to emotions can be a contribution to better understand the nature of the emotions that are experienced daily and, at the same time, contribute to the understanding of the semantic content associated with the concepts. The main argument is that, on the one hand, the presence of a prototypical perceptual referent is considered one of the most important factors when differentiating between concrete and abstract concepts, and on the other hand, prototypical facial expressions are considered a determining factor in differentiating between basic emotions and secondary emotions. In this way, the purpose of this study has been to investigate the role that prototypical facial expressions can have in the quantitative differentiation between basic and secondary emotions in the perception of concreteness and, in addition, if there are qualitative differences at the level of attributes associated with these types of concepts. Next, some relevant theoretical and empirical aspects are synthesized regarding the differences between concrete and abstract concepts, regarding basic and secondary emotions and finally, regarding the perception of concreteness, imaginability, availability of context and the analysis of the attributes of the concept.

### Abstract concepts and concepts of emotions

Concepts are basic units of cognition and play a fundamental role in basic psychological processes and in understanding the experiences that occur in the social world (Niedenthal, 2008; Hoemann and Barrett, 2018). Thus, we can understand concepts as mental representations of categories of (natural and artificial) entities, situations, experiences, and actions (Niedenthal, 2008; Cavanaugh et al., 2015) and as mental entities that provide factual knowledge by integrating our sensory and motor experiences with the environment (Humphreys et al., 1988; Kiefer and Pulvermüller, 2012; Harpaintner et al., 2018). Furthermore, concepts are critical to explaining how people classify objects and make generalizations (Hernández, 2017). When a concept alludes to an object where clear physical referents that can be perceived through the senses are identified, it is referred to as a concrete concept (e.g., table), on the contrary, when a concept refers to something that does not have clear physical correlates identifiable by the senses, it is referred to as an abstract concept (e.g., truth) (Borghi et al., 2017). They were based on sensorimotor experiences, but to a greater extent on linguistic, social, and internal experiences (Borghi, 2020, 2022).

Abstract concepts constitute a very heterogeneous group, including those related to mental states, social phenomena, institutional, mathematical and emotion concepts, and have been the topic of several important studies (Setti and Caramelli, 2005; Crutch et al., 2013; Ghio et al., 2013; Roversi et al., 2013; Barca et al., 2017; Harpaintner et al., 2018; Mazzuca et al., 2018; Villani et al., 2019). Although it is important to point out that all concepts have to some extent an emotional charge (Altarriba and Basnight-Brown, 2011; Wu et al., 2021), in the case of concepts referring to emotions, these refer to emotional states such as anger or joy (Altarriba et al., 1999; Altarriba and Bauer, 2004; Xu et al., 2017) and are developed throughout life through social interactions that occur in different contexts, in which language plays an important role (Hoemann et al., 2019) just as abstract concepts (Fini et al., 2021).

In this regard, concepts referring to emotions (e.g., anger, joy, shame) represent an interesting case study because there is debate as to whether they constitute a subset of abstract concepts (Kousta et al., 2011), or rather, form a separate category. For example, emotion words are better remembered than concrete and abstract words and rank differently from concrete and abstract concepts in concreteness, imageability, and context availability (Schwanenflugel et al., 1992). They are also processed faster than other abstract concept words, regardless of their polarity (Altarriba et al., 1999; Altarriba and Bauer, 2004; Kousta et al., 2009). Along these lines, recent studies (Barca et al., 2017; Mazzuca et al., 2018) support the idea that emotion concepts differ from other non-emotional abstract concepts (Altarriba et al., 1999; Altarriba and Bauer, 2004; Setti and Caramelli, 2005). Moreover, from the point of view of embodied theory, it is much easier to show that emotion concepts, unlike abstract concepts, activate various bodily sensations and are based on sensorimotor and emotional systems (Borghi and Binkofski, 2014) therefore, facial expressions could play a relevant role in their differentiation.

In general, an emotional episode can provide the person with information from at least three different sources. The subjective feeling of emotion, physiological changes and facial expressions, postural changes and changes in the intonation of the voice. The first two are experienced in the first person and the third are observable in others (Buck, 1999). A concept of emotion should include these three types of sensory information in an integrated way, along with cultural knowledge mediated by language. About, traditional models propose that concepts are represented in the brain amodally, that is, symbolically and separately from the sensory and motor systems. These models assume that sensory and motor brain systems are not causally related to the retrieval of conceptual information (McClelland and Rogers, 2003) and allow the representation of both concrete and abstract concepts to be explained naturally (Harpaintner et al., 2018). In contrast, more recent theories of embodied or situated cognition (Gallese and Lakoff, 2005; Pulvermüller, 2005; Barsalou, 2008; Borghi and Cimatti, 2009; Kousta et al., 2009; Louwerse, 2011; Meteyard et al., 2012; Kiefer and Barsalou, 2013), propose that both concrete and abstract concepts are represented in specific brain areas associated with different processing modalities. These theories propose that the different characteristics of concepts are represented through cellular assemblies distributed in different sensory, motor, introspective and emotional brain regions primarily associated with the experience associated with the interaction with the concept’s referent. From individual experience, neural networks are activated and consolidated around the verbal label of the concept and can be activated from any of its nodes (Kiefer et al., 2007; Beilock et al., 2008; Lyons et al., 2010; Willems et al., 2010; Hoenig et al., 2011). Recently, hybrid models have also been proposed, which suggest that conceptual knowledge is the result of processing in modality-specific brain circuits interacting with multimodal connection centers (Kiefer and Pulvermüller, 2012; Garagnani and Pulvermüller, 2016). The latter are thought to serve general semantic binding and integration. From these embodied perspectives, and as previously proposed, prototypical facial expressions could form an important node in networks of neural activity associated with a basic emotion concept.

### Theories of emotions and facial expressions

We consider it important to also include a review of two theories that have different proposals regarding what an emotion is. This is because it is important to consider what kind of object or event the concepts of emotion refer to and the role of facial expressions. Neurocultural theory (Ekman, 1999) points out that prototypical facial expressions are essential for differences between basic and secondary emotions and in the same way, facial expressions could constitute a perceptual aspect that would determine a more specific perception of basic emotions with respect to secondary emotions, analogous to the role played by perceptual aspects in the differentiation between concrete and abstract concepts. Posits the existence of basic, discrete, universal emotions, with a strong biological component, which are independent of language and culture (Ekman and Friesen, 1971; Anguas-Wong and Matsumoto, 2007; Matsumoto and Willingham, 2009; Ekman and Cordaro, 2011; Jack et al., 2012). This theory conceives of emotions as natural types, that is, biological devices of preconfigured physiological and muscular-expressive responses to a set of stimuli that have phylogenetic adaptive value. It is based mainly on evidence of universality for seven basic emotions: anger, fear, sadness, surprise, disgust, contempt and joy, and each of them would be associated with a phylogenetically inherited physiological pattern and prototypical facial expressions that can be identified and recognized by any person independent of their culture (Ekman, 1994, 1999; Ekman and Cordaro, 2011). Thus, when a person experiences an emotion, a specific effector pattern of that emotion is activated and communicated to others through a distinctive facial expression. When there is no prototypical facial expression, the emotion would not be basic, but secondary (Ekman, 1993, 1999; Ekman and Cordaro, 2011). Additionally, facial-muscular activity sends neural feedback to the brain that specifies the quality or nature of the feeling of emotion being experienced (Ekman, 1999; Ekman and Cordaro, 2011).

On the other hand, there are theories that conceive emotions as situated psychological constructs, which are consistent with theories of embodied cognition (Vytal and Hamann, 2010; Gendron et al., 2014; Siegel et al., 2018; Sato et al., 2019). Among the more recent of these theories, conceptual act theory (Barrett, 2009, 2012, 2014, 2017) assigns a preponderant role to concepts of emotion (Hoemann and Barrett, 2018). In conceptual act theory (Barrett, 2009), it is proposed that cognitive processes and affective processes emerge from the same set of basic brain-level elements (Russell, 2003; Kuppens et al., 2012): appraisal or affect (change in a person’s internal physical state that can be consciously experienced as pleasant or unpleasant; Moors et al., 2013), categorization (determining what something is, why it is, and what to do about it), and attention (anything that can change the rate of neural activation) (Barrett, 2009, 2006, 2017). Regarding emotions, conceptual act theory posits that emotions are constructed, corresponding to phenomena that only exist and acquire meaning in the social/cultural domain and not as natural types (Barrett, 2012). The theory proposes that physical changes internal to the subject and changes in the external world (actions of others, physical environment, etc.) become real as emotion when they are categorized as such. For this, the perceiver uses knowledge of the concept of emotion that they have learned through language and socialization (Barrett, 2017). Categorization is understood as a “situated conceptualization,” meaning that the conceptual knowledge that is applied is linked to the context in which it occurs (Barrett, 2014). Thus, for this theory, facial expressions play a marginal role, since, without emotional concepts there are only faces that arbitrarily move (Barrett, 2017).

Another relevant aspect of emotions is that they are made up of different components that are causally related to each other. An event/stimulus that interacts with an appraisal component, which in turn activates the physiological, motivational, and expressive components, which together specify the subjective feeling of the emotion, which is verbally categorized (Meuleman et al., 2019). It is important to add that emotions are triggered by stimuli or events that are adaptively relevant to people. When a specific emotion is activated, effector patterns are triggered that biologically and cognitively configure the organism to select the response that best adapts to the context, as is the case of approach avoidance responses to positive and negative stimuli (Fini et al., 2020). However, it is not yet clear if these components are reflected in social knowledge about emotions and the semantic content that people have regarding emotion concepts.

### Perception of concreteness and attributes of concepts

One approach to the study of the concepts has been in terms of the subjective perception of three qualities: *concreteness*, or the degree to which the concept refers to a perceivable entity (Brysbaert et al., 2014); *imageability*, or the effectiveness with which a word triggers a mental image (Rofes et al., 2017); and *context availability*, or the possibility of accessing contextual information relating to the concept (Taylor et al., 2018). The notion of concreteness has been studied from the perspective of the Dual Coding Theory (Paivio, 1986, 2013), which proposes the existence of two independent but interconnected representational systems: a verbal system (the logogen system) and an image generation system (the imagen system). Both concrete and abstract concepts are represented in the logogen system, but only concrete concepts are connected to the imagen system. This makes concrete concepts easier to recollect. The degree of imageability of a concept is based on the above premise: the more sensory-motor information is associated with a word (imagen system), the easier that word is to imagine (Westbury et al., 2016). The notion of context availability (Rofes et al., 2017) suggests that it is easier to access contextual information for concrete words (which tend to be associated with more specific contexts) than for abstract words (which tend to be associated with a variety of contexts). About, Altarriba and Bauer (2004) found that emotion concepts are perceived differently to abstract and concrete concepts on each of the three assessment scales but did not study the differences between basic and secondary emotions.

Another approach to the study of concepts has been through their attributes. The attributes of a concept are those properties, qualities or characteristics that are considered unique to an object, situation or person and account for the semantic content associated with the concept. An alternative for the study of concept attributes is the property listing task (McRae et al., 2005; Devereux et al., 2013; Chaigneau et al., 2017), which consists of asking a person to write a list of each of the attributes that they consider to be associated with a specific concept that is presented as a stimulus. Attribute listings have been used to create conceptual property norms, which have provided information on numerous phenomena related to concept processing, categorization, and semantic memory, and have been used to test theories and models and to study inter- and intra-subjective variability (McRae et al., 2005; Chaigneau et al., 2017).

Using the property listing task Barsalou and Wiemer-Hastings (2005), found that for concrete concepts, properties related to objects, locations, and behaviors in situations predominated, whereas for abstract concepts, introspections, mental states, and social aspects of situations with intermediate concepts in between predominated. Later studies additionally applied the hierarchical cluster analysis technique and found that abstract concepts are more strongly associated with emotions, social cognition, internal and mental states than concrete concepts (Troche et al., 2014, 2017; Binder et al., 2016). Harpaintner et al. (2018) coded property listings using a scheme adopted from Barsalou and Wiemer-Hastings (2005) and through a cluster analysis revealed different subcategories of abstract concepts showing that these are very heterogeneous and that the investigation of well-defined subcategories of these concepts is required.

An aspect still little studied regarding the attributes of emotion concepts refers to the way in which people use the knowledge they have about emotions to integrate the different types of components of the emotional episode and the different aspects of the situation or context (Barrett, 2017). Considering that the emotional episode follows a temporal sequence of cause and effect relationships and considering the importance of emotions for social adaptation, it is proposed that the cause-effect scheme could be an important category to classify the attributes listed for the emotion concepts. And that would allow them to be differentiated from concepts not related to emotions. The semantic study of this type of concepts will allow a better understanding of how people conceptualize, understand and attribute meaning to the emotions and sensations that emanate from their own body (Zammuner, 1998).

Thus, the objective of the present study was to determine if facial expressions allow us to differentiate between basic and secondary emotion concepts and if there are differences in the type of semantic content between specific emotion concepts and abstract concepts that do not describe prototypical emotions, using the tasks of perception of concreteness, imageability and availability of context and the task of the list of properties.

## Materials and methods

### Design

A repeated measures and complex factorial design (2 × 2) was employed. The first independent variable was called “perceptual referent” and consisted of the possibility of identifying (or not) clearer or prototypical salient perceptual elements related to the concept, for example, “tree” versus “idea” or “anger” (having a prototypical facial expression) versus “hope.” This does not imply that there are no perceptual aspects for abstract concepts or secondary emotions, it is only stated that if they do exist, they would not be prototypical of the concept. The second independent variable was called “concept type” and differentiated whether the referent of the concept is an emotion or an object not related to an emotion. Each variable has only two levels and, in this way, four conditions are created from the independent variables by combining the two levels of each: (i) basic emotion concepts (e.g., joy, disgust, fear), that groups concepts with some evident perceptual element and refers to emotions, (ii) secondary emotion concepts (e.g., compassion, relief, guilt), that groups concepts related to emotions but without an evident perceptual aspect, (iii) concrete non-emotion concepts (e.g., tree, scissors, table), that groups concepts not related to emotions and with a more salient perceptual referent, and (iv) abstract non-emotion concepts (e.g., freedom, truth, decency) that groups concepts not related to emotions and without an evident perceptual referent. In the proposed design it is assumed that the basic emotion concepts and the concrete concepts share common elements, which would be the presence of clearer, more salient, more evident perceptual referents or greater cultural consensus. Emotion-related concepts were differentiated into basic and secondary emotions based on neurocultural theory (Ekman, 1999) and non-emotion-related concepts were differentiated into abstract (e.g., freedom) and concrete (e.g., table).

The dependent variables correspond to the performance in rating studies along different dimensions and the listing properties task, and from them different indices are elaborated that will be explained later. An independent statistical analysis will be carried out for each of the indexes indicated.

### Participants

Eighty-six university students from the conurbation La Serena - Coquimbo (Chile), all native speakers of Spanish, were selected non-randomly by snowball sampling to participate in the study. The sample size was defined using Gpower 3.1 (Faul et al., 2007), employing a repeated measures ANOVA analysis of one group and four measurements, while assuming an effect size $\eta_{P}^{2}$ = 0.20, α = 0.05, 1-β = 0.95 and attrition equal to zero. The sample obtained was 55 participants, but a larger number was considered in the eventuality of loss of information. Forty-four percent of the participants were male (*M* = 20 years; *SD* = 2.19) and 56% were female (*M* = 20 years; *SD* = 2.92). The participants’ fields of study were: 19%—health and psychology, 37%—education, 27%—engineering, and 17%—other fields.

### Procedure

First, the concepts (stimuli) were rigorously selected according to the research design. That they were in common use in Spanish and that they reflected the heterogeneity of both concrete and abstract concepts. Regarding the concepts of secondary emotions, those that clearly did not have a prototypical facial expression were chosen. Regarding the concepts referring to emotions, the basic emotions of neurocultural theory (Ekman, 1999) and secondary emotions from the Lazarus and Lazarus (2000) classification were included. As for the concepts not referring to emotions, the concrete and abstract concepts reported in the experiment by Altarriba and Bauer (2004) were considered. The concepts of basic emotions were: joy, sadness, anger, disgust, fear, surprise, contempt, and fury. The secondary emotions were: love, melancholy, guilt, anxiety, relief, compassion, pride, and envy. The concrete concepts were: tree, table, cloud, airplane, machine, scissors, dragon, and movie. And the abstract concepts were: commitment, truth, freedom, attitude, teaching, excuse, fiction, and essence (see Table 1). The basic emotion concepts numbered seven, and this determined the total number of concepts per condition. A synonym for anger (fury) was added to bring the total concepts to eight for each condition. In the selection of the other concepts, we try to include concepts that are more frequently used in the cultural context and at the same time represent its heterogeneity (for example, dragon, film, and tree). In addition, an attempt was made to include concepts of positive and negative valence, although there was a difficulty because the cases of basic emotions are mostly negative. An alternative was to include only concepts with a negative valence in all conditions, however it was decided to include concepts with both types of valences. Finally, the task instructions were translated into Spanish and evaluated by a panel of experts. When the words for this study were selected, the study by Pérez-Sánchez et al. (2021) had not yet been published with norms for emotion words in Spanish, however, Tables 2, 3 include information on prototypicity, valence, concreteness and other relevant parameters. In addition, the concreteness averages for each of the words selected for this study are included. Table 2 shows the standards reported by Pérez-Sánchez et al. (2021), for the emotion words used in this study. It is observed that the words of basic emotion have a higher average of prototypicity and arousal, but not of valence, than those of secondary emotions. The concretion norms are also shown, and the concretion data obtained in the present study were added for each of the words. In this regard, a decrease in the perceived concreteness is observed. Table 3 shows the valence and arousal norms for words not referring to emotions concrete and abstract, reported by Stadthagen-Gonzalez et al. (2017). It should be considered that the prototypicity norms used a scale from 1 to 5, while in valence and arousal, a scale from 1 to 9.

**TABLE 1:** Selected concepts in the present study.

Perceptual prototypical referent		Non-prototypical referent	
*Emotion*	*No emotion*	*Emotion*	*No emotion*
Alegría (joy)	Árbol(tree)	Amor (love)	Compromiso (commitment)
Tristeza (sadness)	Mesa (table)	Melancolía (melancholy)	Verdad (truth)
Rabia (anger)	Nube (cloud)	Culpa (guilt)	Libertad (freedom)
Asco (disgust)	Avión (airplane)	Ansiedad (anxiety)	Actitud (attitude)
Miedo (fear)	Máquina (machine)	Alivio (relief)	Docencia (teaching)
Sorpresa (surprise)	Tijeras (scissors)	Compasión (compassion)	Excusa (excuse)
Desprecio (contempt)	Dragón (dragon)	Orgullo (pride)	Ficción (fiction)
Furia (fury)	Película (movie)	Envidia (envy)	Esencia (essence)

**TABLE 2:** Norms provided by Pérez-Sánchez et al. (2021) for the emotion words and concreteness included in the present study.

Word	Prototypicality		Valence		Arousal		Concreteness		Concreteness in this study		Emotionality	Dominant emotion
*Words basic emotion*	Mean	SD	Mean	SD	Mean	SD	Mean	SD	Mean	SD	Mean	
Alegría (joy)	4.95	0.22	8.35	1.69	6.75	2.51	4.08	#NA	**3.94**	2.12	3.35	Happiness
Tristeza (sadness)	4.91	0.36	1.80	1.51	4.45	2.37	4.35	#NA	3.74	2.07	3.20	Sadness
Rabia (anger)	4.90	0.30	2.65	1.35	7.60	1.37	4.76	#NA	3.57	1.97	2.35	Anger
Asco (disgust)	4.14	1.42	2.55	1.19	6.65	1.09	4.53	#NA	3.85	1.88	2.45	Disgust
Miedo (fear)	4.90	0.30	2.00	1.17	8.00	1.49	3.43	#NA	3.59	2.09	3.00	Fear
Sorpresa (surprise)	4.43	1.03	7.75	1.07	7.40	1.67	4.21	#NA	3.89	1.94	2.75	Happiness
Desprecio (contempt)	3.65	1.42	2.35	1.04	6.25	1.92	4.56	1.71	3.26	1.85	2.65	Anger
Furia (fury)	4.05	1.60	3.15	1.87	8.05	1.10	4.72	1.65	3.52	1.85	1.85	Anger
Mean	4.49		3.83		6.89		4.33		3.67		2.70	
***Words secundary emotion***												
Amor (love)	3.99	1.24	8.40	1.03	6.40	3.05	2.59	#NA	3.57	2.44	3.40	Happiness
Melancolía (melancholy)	4.05	1.17	2.85	1.39	5.00	2.51	4.66	#NA	3.16	1.70	2.15	Sadness
Culpa (guilt)	3.59	1.47	2.55	1.05	6.95	1.10	3.62	#NA	3.18	1.92	2.45	Sadness
Ansiedad (anxiety)	3.67	1.53	2.10	0.91	7.70	1.22	4.48	1.83	3.70	1.87	2.90	Fear
Alivio (relief)	3.38	1.60	8.00	0.92	2.95	1.96	3.78	#NA	3.56	1.90	3.00	Happiness
Compasión (compassion)	3.38	1.60	5.75	1.89	3.70	1.22	3.16	#NA	3.29	1.81	0.75	Sadness
Orgullo (pride)	3.76	1.14	4.95	2.30	6.50	1.97	5.22	#NA	3.41	1.96	0.05	Anger
Envidia (envy)	4.00	1.30	2.35	0.99	7.10	1.33	4.27	#NA	3.31	1.77	2.65	Anger
Mean	3.73		4.62		5.79		3.97		3.40		2.17	

Prototypicality, minimum score 1 and maximum 5; valence and arousal, minimum score 1 and maximum 9; concreteness, minimum score 1 and maximum 7.

**TABLE 3:** Valencia and arousal norms provided by Stadthagen-Gonzalez et al. (2017) for words not referring to emotion used in the present study.

	Valence		Arousal			Valence		Arousal	
*Words no emotion concrete*	Mean	SD	Mean	SD	*Words no emotion abstract*	Mean	SD	Mean	SD
Árbol(tree)	6.8	1.5	3.6	1.6	Compromiso (commitment)	6.3	1.9	6.3	1.8
Mesa (table)	5.2	0.8	4.7	1.0	Verdad (truth)	7.6	1.4	4.1	2.5
Nube (cloud)	6.9	1.3	3.6	1.9	Libertad (freedom)	8.6	0.7	4.7	2.8
Avión (airplane)	6.5	1.4	6.4	1.6	Actitud (attitude)	6.5	1.7	5.4	1.8
Máquina (machine)	5.5	1.0	5.2	1.3	Docencia (teaching)	6.5	1.2	4.8	1.1
Tijeras (scissors)	4.8	0.9	5.3	1.5	Excusa (excuse)	2.7	1.4	5.9	1.5
Dragón (dragon)	5.7	1.8	6.0	2.2	Ficción (fiction)	6.2	1.9	5.7	1.6
Película (movie)	7.7	1.3	4.9	2.2	Esencia (essence)	7.3	1.4	4.7	2.5
Mean	6.1		4.9		Mean	6.4		6.1	

Valence and arousal, minimum score 1 and maximum 9.

The indexes for data analysis in the property listing task emerged from the theoretical analysis and were validated by expert linguists. The main criterion used was that they allowed a clear and relevant classification of the listed properties. Subsequently, scores were assigned and the percentage of agreement between two raters scoring the same random selection of responses was calculated. The raters did not know the objectives of the experiment. The percentages of agreement achieved were 80% for the “total number of attributes” index, 84% for the “number of causal attributes” index. The Kappa coefficient was not used due to the presence of constant values and identical agreement between raters, which made it unsuitable (Delgado et al., 2020).

In relation to the collection phase, snowball sampling was used whereby potential participants were contacted by sending them a link to respond remotely on a Google form (this measure had to be adopted due to the COVID-19 pandemic). Before responding, individuals had to read the online informed consent form and check the “Yes” box to agree to participate. Before being administered, this task was approved by the Ethics Committee of the University of La Serena (Chile). Firstly, the task of perception of concreteness, imageability and availability of context was presented, and secondly, the task of listing properties.

### Experimental task

#### Rating of concreteness, imageability, and context availability

For each concept, participants were asked to assign a score from 1 to 7 on each of the following three scales: concreteness (1: *highly abstract*, 7: *highly concrete*), imageability (1: *difficult to imagine*, 7: *easy to imagine*), and context availability (1: *difficult to think about in a context*, 7: *easy to think about in a context*). On each scale, a higher score implies greater perception of concreteness, imageability, and context availability. The concepts (stimuli) were presented sequentially and randomly to each participant. The final score was obtained by adding the responses to the stimuli within each of the four conditions (concepts related to basic emotions; concepts related to secondary emotions; concrete non-emotional concepts; abstract non-emotional concepts).

### Property listing task

This task is used to investigate semantic content based on the properties that people assign to different types of concepts (Chaigneau et al., 2017). Participants are asked to write lists of properties or characteristics that describe a specific concept. From the responses, different indices can be created. For the present research, the following indices were used:

Index 1 - Total number of properties listed: Corresponds to the total frequency of properties listed in each of the four groups formed by the experimental conditions. Those responses that do not correspond to attributes were not considered. One point was assigned for each attribute and the total was added up.

Index 2 - Specific number of cause-and-effect properties: Among the different types of properties listed, we identified those attributes that refer to causal relationships (e.g., tree; produces oxygen; anger: makes you want to attack). That is, that the concept presented is seen as a cause or as an effect in a temporal sequence. One point was assigned to each cause-and-effect property and the total was added up (the other types of attributes were not considered).

### Data analysis

For the data analysis, a Linear General Model of repeated measures, ANOVA test was employed together with partial eta squared to know the effects of each of the experimental conditions on the different indexes of the dependent variable. This makes it possible to evaluate the effect of the independent variables, each one separately, and also the interaction effect of both together, on a dependent variable (a separate analysis is performed for each dependent variable). All these analyses were carried out using Jamovi 1.6 software.

## Results

### Perception of concreteness

Regarding the effect of the type of concept on the perception of concreteness, a significant effect was found [*F*(1,85) = 79.80, *p* < 0.001, $\eta_{P}^{2}$ = 0.484) such that the concepts referring to emotion (M = 28.51; SE = 1.31) were perceived as less concrete (more abstract) than the concepts not referring to emotion (M = 37.54; SE = 0.78). In turn, the perceptual referent also presented a significant effect (F(1, 85) = 134.14, p < 0.001, n2p = 0.612) so that the concepts with a more salient perceptual referent (M = 38.37; SE = 0.84) they were perceived as more concrete than those without a clear perceptual referent (M = 27.69; SE = 1.25).

**FIGURE 1:**
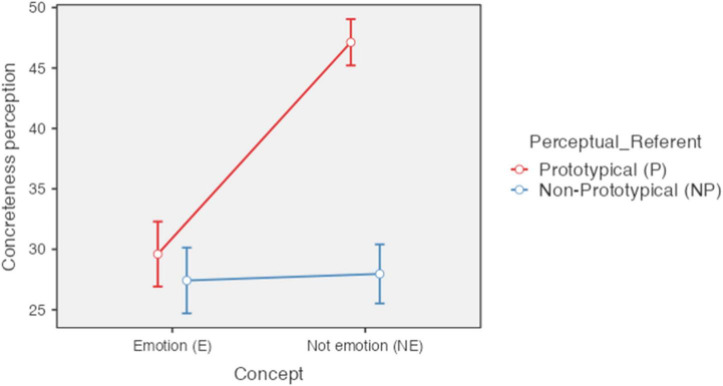
Perception of concreteness according to type of concept and type of perceptual referent. Error bars 95% CI; significant differences between concepts of, basic emotion and concrete (E-P_NE-P>; secondary emotion and concrete (E-NP_NE-P); concrete and abstract (NE-P_NE-NP). *P* < 0.001. Created with Jamovi 1.6.

The interaction effect between the type of concept and the perceptual referent allows a more detailed analysis of the means obtained for each of the four experimental conditions: concepts referring to emotion with perceptual referent (basic emotions); concepts referring to emotion without perceptual referent (secondary emotions); concept not referring to emotion with perceptual referent (concrete); and concept not referring to emotion without perceptual referent (abstract). In this case, the interaction effect was significant [*F*(1,85) = 79.80, *p* < 0.001, $\eta_{P}^{2}$ = 0.484). Means for perception of concreteness were, for concepts referring to emotion with perceptual referent *M* = 29.60 (*SD* = 12.54), for concepts referring to emotion without perceptual referent *M* = 27.42 (*SD* = 12.69), for non-emotion concepts with perceptual referent *M* = 47.13 (*SD* = 8.93), and for non-emotion concepts without perceptual referent *M* = 27.97 (*SD* = 11.40) (see Figure 1).

The *post hoc* analysis revealed no significant difference in perception of concreteness between concepts referring to emotion with perceptual referent and without perceptual referent [*t*(168) = 1.793, *pbonferroni* = 0.449], between concepts of emotion and non-emotion without perceptual referents [*t*(170) = 1.130, *pbonferroni* = 1], and between concepts of emotion with perceptual referent and non-emotion without perceptual referents [*t*(161) = −0.474, *pbonferroni* = 1]. However, a significant difference was found between concepts referring to emotion with perceptual referent and concepts not referring to emotion with perceptual referent [*t*(161) = −13.912, *pbonferroni* < 0.001), between concepts referring to emotion without perceptual referent and concept not referring to emotion with perceptual referent [*t*(170) = −14.588, *pbonferroni* < 0.001), and between concept not referring to emotion with perceptual referent and concept not referring to emotion without perceptual referent [*t*(168) = 16.117, *pbonferroni* < 0.001] (see Figure 1). In summary, both concepts referring to emotion and abstract concepts were perceived in a similar way and very different from the concrete concepts.

### Perception of imageability

**FIGURE 2:**
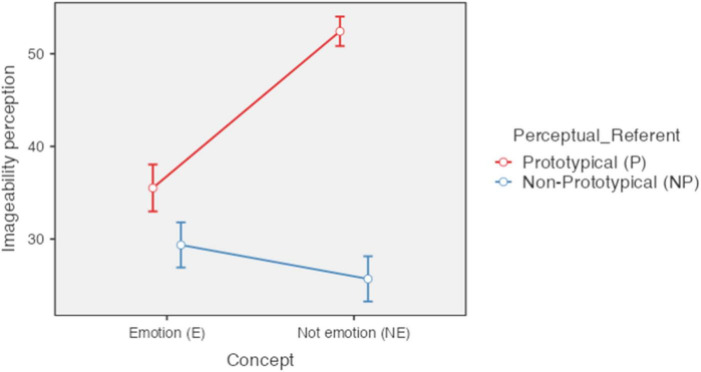
Perception of imageability according to type of concept and type of perceptual referent. Error bars 95% CI; significant differences between concepts of, basic and secondary emotion (E-P_E-NP); basic emotion and concrete (E-P_E-NP); basic emotion and abstract (E-P_NE-NP; secondary emotion and concrete (E-NP_NE-P). *P* < 0.001. Created with Jamovi 1.6.

A significant effect was found for concept type [*F*(1,85) = 61.83, *p* < 0.001, $\eta_{P}^{2}$ = 0.419], perceptual referent [*F*(1,85) = 304.37, *p* < 0.001, $\eta_{P}^{2}$ = 0.782] and the interaction between both [*F*(1,85) = 241.17, *p* < 0.001, $\eta_{P}^{2}$ = 0.739]. The concepts related to emotion (*M* = 32.44; *SE* = 1.18) were considered more difficult to imagine than those not related to emotion (*M* = 39.06; *SE* = 0.78) and the concepts with a referent (*M* = 39.06; SE = 0.78) were they considered easier to imagine than those without reference (*M* = 39.06; *SE* = 0.78).About the interaction, means for perception of imageability were, for concepts referring to emotion with perceptual referent *M* = 35.51 (*SD* = 11.81), for concepts referring to emotion without perceptual referent *M* = 29.36 (*SD* = 11.37), for non-emotion concepts with perceptual referent *M* = 52.42 (*SD* = 7.41), and for non-emotion concepts without perceptual referent *M* = 25.70 (*SD* = 11.40).The *post hoc* analysis revealed that the only difference that was not significant occurred between the concepts without a perceptual referent referred to and not referred to emotion [*t*(162) = 3.27, *pbonferroni* = 0.008]. All other comparisons were significant (*p* < 0.01) (see Figure 2).

### Context availability

**FIGURE 3:**
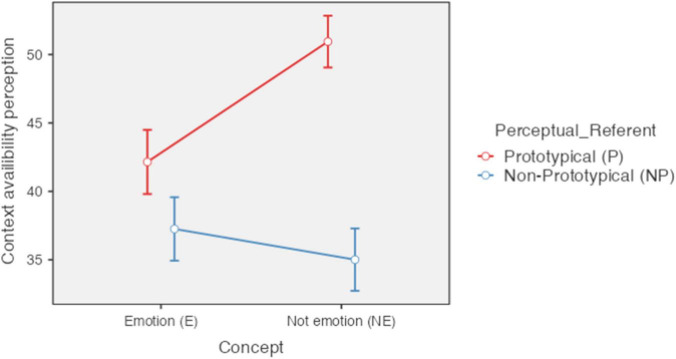
Context availability perception according to type of concept and type of perceptual referent. Error bars 95% CI; significant differences between concepts of, basic and secondary emotions (E-P_E-NP); basic emotion and concrete (E-P_NE-P); basic emotion and abstract (E-P_NE-NP); secondary emotion and concrete [E-NP_NE-P); secondary emotion and abstract (E-NP_NE-NP); concrete and abstract (NE-P_NE-NP). *P* < 0.001. Created with Jamovi 1.6.

A significant effect was found for concept type [*F*(1,85) = 19.47, *p* < 0.001, $\eta_{P}^{2}$ = 0.186), perceptual referent [*F*(1,85) = 155.33, *p* < 0.001, $\eta_{P}^{2}$ = 0.646) and the interaction between both [*F*(1,85) = 87.95, *p* < 0.001, $\eta_{P}^{2}$ = 0.509). The concepts related to emotions (*M* = 39.70; *SE* = 1.12) were considered to have less context availability than those not related to emotions (*M* = 42.98; *SE* = 0.84) and the concepts with a referent (*M* = 46.55; *SE* = 0.91) were they considered with more context availability than those without reference (*M* = 36.13; *SE* = 1.10). About the interaction, means for perception of imageability were, for concepts referring to emotion with perceptual referent *M* = 42.15 (*SD* = 10.93), for concepts referring to emotion without perceptual referent *M* = 37.26 (*SD* = 10.82), for non-emotion concepts with perceptual referent *M* = 50.94 (*SD* = 8.82), and for non-emotion concepts without perceptual referent *M* = 35.01 (*SD* = 10.62). In the *post hoc* analysis it is observed that the only difference that was not significant occurred between the concepts without a perceptual referent referred to and not referred to emotions [*t*(163) = 2.18, *pbonferroni* = 0.183]. All other comparisons were significant (*p* < 0.01) (see Figure 3).

### Properties

The results show that concepts of emotion (*M* = 21.94; *SE* = 0.80) presented a lower average frequency than non-emotion concepts (*M* = 23.89; *SE* = 0.88) [*F*(1,85) = 29, *p* < 0.001, $\eta_{P}^{2}$ = 0.254). On the other hand, concepts with prototypical perceptual referents (*M* = 25.19; *SE* = 0.95), such as basic emotion concepts and concrete concepts, had a higher frequency of properties than concepts with non-prototypical referents (*M* = 20.64; *SE* = 0.75) [*F*(1,85) = 92.1, *p* < 0.001, $\eta_{P}^{2}$ = 0.520). In addition, both variables were found to have an interaction effect [*F*(1,85) = 145.9, *p* < 0.001, $\eta_{P}^{2}$ = 0.632].

**FIGURE 4:**
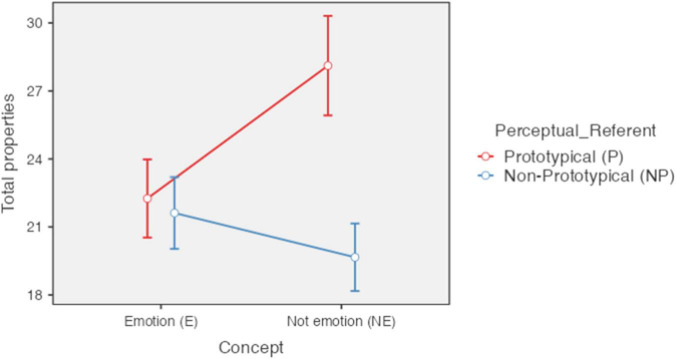
Number of properties by type of concept and clarity of the perceptual referent. Error bars 95% CI; significant differences between concepts of, basic emotion and concrete (E-P_NE-P); basic emotion and abstract (E-P_NE-NP); secondary emotion and concrete (E-NP_NE-P);secondary emotion and abstract (E-NP_NE-NP); concrete and abstract (NE-P_NE-NP). *P* < 0.801. Created with Jamovi 1.6.

As shown in Figure 4, the highest number of properties was obtained by the concrete concepts not referring to emotion (e.g., airplane) (*M* = 28.1; *SD* = 0.89) followed by the concepts of basic emotions (e.g., fear) (*M* = 22.3; *SD* = 0.89), with a significant difference between them [*t*(168) = -12.05, *pbonferroni* = 0.001]. Then, with a lower number were secondary emotion concepts (e.g., shame) (*M* = 21.6; *SD* = 0.89) followed by non-emotion abstract concepts (*M* = 19.7; *SD* = 0.89) which presented the lowest number. The difference in means between these two types of concepts was also significant [*t(*168) = 4.02, *pbonferroni* < 0.001]. All other mean comparisons were significant with the exception of basic (*M* = 22.3; *SD* = 0.89) and secondary (*M* = 21.6; *SD* = 0.89) emotion concepts [*t*(150) = 1.11, *pbonferroni* = 1].

### Cause-effect properties

The emotion concepts (*M* = 11.56; *SE* = 0.24), in general, present a greater amount of cause-and-effect properties than non-emotion concepts (*M* = 8.59; *SE* = 0.08) [*F*(1,85) = 187.2, *p* < 0.001, $\eta_{P}^{2}$ = 0.688]. It is also apparent that on average concepts with perceptual referents (*M* = 10.29; *SE* = 0.15) presented more cause-and-effect properties than concepts without a prototypical referent (*M* = 9.86; *SE* = 0.16), [*F*(1,85) = 12.9, *p* < 0.001, $\eta_{P}^{2}$ = 0.132]. In addition, an interaction effect between both variables is observed [*F*(1,85) = 27.4, *p* < 0.001, $\eta_{P}^{2}$ = 0.244).

**FIGURE 5:**
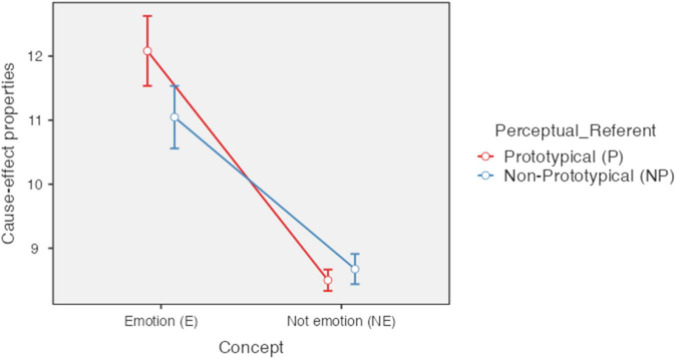
Number of cause-and-effect properties according to type of concept and type of perceptual referent. Error bars 95% CI; Significant differences between concepts of, basic and secondary emotion (E-P_E-NP); basic emotion and concrete (E-P_NE-P); basic emotion and abstract (E-P_NE-NP); secondary emotion and concrete (E-NP_NE-P); secondary emotion and abstract (E-NP_NE-NP). *P* < 0.001. Created with Jamovi 1.6.

As shown in Figure 5, the highest average of cause-and-effect properties corresponds to the concepts with prototypical referent (basic emotions) (*M* = 4.12; *SD* = 0.273) followed by the concepts without prototypical referent (secondary emotions) (*M* = 3.07; *SD* = 0.246) and the difference between them was significant [*t*(85) = 5.26, *pbonferroni* < 0.001]. With a lower average are the concepts not referring to emotion without prototypical referent (abstract concepts) (*M* = 0.71; *SD* = 0.114) and finally the concepts not referring to emotion with prototypical referent (concrete concepts), which presented the lowest average of all (*M* = 0.52; *SD* = 0.081). As can be seen, the difference between these last two averages was not significant [*t*(85) = −1.43, *pbonferroni* = 0.931]. All other comparisons related to Figure 2 were significant. In summary, the emotion concepts, and in particular the basic emotions, presented a greater number of cause-and-effect properties than the non-emotion concepts.

## Discussion

A significant effect of perceptual referent was found. Taken together, concepts with a perceptual referent (basic emotions and concrete) were perceived as more concrete, easier to imagine, and with greater availability of context than concepts without an obvious perceptual referent (abstract and secondary emotions). In the property listing task, concepts with a perceptual referent generated a greater number of total attributes and a greater number of cause-effect type attributes. Also, a significant effect of concept type was found. Emotion concepts (basic and secondary) are perceived as less concrete, more difficult to imagine, and more difficult to place in context than non-emotional concepts (concrete and abstract) taken together. Similarly, emotional concepts generate fewer total attributes but producing more cause-effect type attributes.

These results, without considering the interaction effect between the two variables, show that the presence or absence of a prototypical perceptual referent can be an important factor to explain the differences in the perception of concreteness, imageability and availability of context, between concrete and abstract concepts, as various authors have pointed out (Borghi et al., 2017, 2022). On the other hand, it is also evident that the concepts of emotions are perceived differently from the concepts not related to emotions, however, to adequately interpret these results, it is necessary to analyze in detail the interaction effect.

Regarding the perception of concreteness, as expected, the concrete concepts were widely perceived with greater concreteness than the non-emotional abstract concepts. However, contrary to expectations, no difference was observed between concepts of basic and secondary emotions, and both were perceived with the same concreteness as abstract non-emotional concepts. According to these results, both concepts of basic and secondary emotions are perceived as abstract as the abstract concepts not related to emotions. This would be inconsistent with the results obtained in the study by Altarriba and Bauer (2004) and Xu et al. (2017). Unlike concreteness, perceived imageability and context availability showed a significant difference between basic and secondary emotion concepts. In addition, basic emotion concepts were easier to imagine and easier to place in context than abstract concepts, whereas secondary emotions did not differ from abstract concepts. These results are consistent with expectations, in the sense that the presence of a prototypical perceptual referent in basic emotions would be facilitating the ability to imagine the concept and place it in a context.

As has been pointed out, the prototypical facial expressions of basic emotions (Ekman, 1993, 1999) are a good alternative to explain the improvement in imageability and availability of context in basic emotions, but not the improvement in the perception of concreteness. This apparent contradiction could be explained by the different sources of location of the components of an emotional episode. The feeling of emotion and other subjective aspects are experienced in themselves and are impossible to observe in others. On the other hand, most of the time it is not possible to observe one’s own facial expression, but that of others. In addition, formal and informal emotion education emphasizes facial expressions of basic emotions (Buck, 1999). For this reason, when rating concreteness, people could pay attention to subjective aspects, but when rating imageability and availability of context, they could focus mostly on facial expressions. According to the conceptual act theory (Barrett, 2009, 2012, 2014, 2017), facial expressions have a marginal role in the conceptualization of emotions and would not be a key factor in differentiating between basic and secondary emotions. Furthermore, at the biological level there would be no solid basis for differentiating between them. The results of this study show that the prototypical perceptual referent of facial expressions at least plays an important role in differentiating between basic and secondary emotion concepts in the dimensions of imageability and context availability.

Taking into account the results of Pérez-Sánchez et al. (2021), basic emotion words that are associated with prototypical facial expressions have higher prototypicality means than secondary emotion words. The authors found that prototypicity of concept is predicted by hedonic load and arousal, which from the perspective of the informative value of emotions is also consistent with the informative role of facial expressions. In the case of hedonic load and arousal, the information is directed at the person experiencing the emotion, and in the case of facial expressions, it is directed at others in a social context, so it is plausible to assume that both aspects, subjective and expressive, are relevant in the conceptualization of emotions.

Regarding valence, as previously indicated, in the selection of basic emotions words negative valence emotions predominate, compared to secondary emotion words and concepts words not referring to emotions. This is corroborated by reviewing the norms of Stadthagen-Gonzalez et al. (2017) and Pérez-Sánchez et al. (2021). Valence has been reported to affect word processing speed and approach/avoidance tendency (Fini et al., 2020), however, in a systematic review on the subject, Kauschke et al. (2019) point out that the studies reviewed show only weak evidence of an advantage in quality or processing speed of words with positive valence over negative ones. They further point out that the valence of most words is idiosyncratic to people and depends on their specific experiences. In this study, the concrete and abstract words, which present the greatest difference between them in the perception of concreteness, have almost similar normative valence averages and the words referring to emotions, which present a slightly greater difference, have a smaller distance in the perception of concreteness. The foregoing allows us to suppose that, at least in the perception of concreteness, there would not be such a marked bias produced by valence. Notwithstanding the foregoing, it cannot be ruled out that the differences in the valences between the different sets of words used in this study may have biased the results, so it would be ideal to replicate the study selecting only words with a negative valence in all conditions.

The results for the total number of properties listed are equivalent to those obtained for the concretion rating. There is no difference between basic and secondary emotions and both types of concepts are not differentiated from abstract non-emotional concepts. The presence of prototypical perceptual referents was expected to facilitate the generation of a greater number of properties for basic emotion concepts than for secondary emotions and abstract concepts. In this dimension of the analysis, the perceptual referents do not seem to play an important role, although they do replicate the differentiation between concrete and abstract concepts. In other words, in terms of property generation, emotion concepts do not differ from abstract concepts. Although the perspective of the Dual-Coding Theory (Paivio, 1986, 2013) makes it possible to clearly explain this effect by proposing an image system and a verbal information processing system, embodied theories state that sensory systems should also be activated when processing abstract concepts, however, it is difficult to determine what perceptual referents might activate sensory systems in the case of those concepts. It is important to consider that in these cases, emotional knowledge related to social interaction processes could be more relevant than conscious or explicit knowledge of the different components of an emotional episode. We consider the latter very relevant, because most people do not consciously experience the different components of the emotional episode, except for the subjective feeling and facial expressions, especially with high levels of activation.

But, when analyzing the frequency of properties associated with a framework of cause-effect relationships, a difference emerges between concepts of basic and secondary emotions and a very clear difference between emotional and non-emotional concepts. In this case, it is more difficult to interpret the role that perceptual referent could have in those results. It is more likely that cultural emotional knowledge and the type of relationship of people with the concepts could better explain the results obtained. The above is relevant because it includes an additional dimension in the analysis of the differences between concepts. When comparing emotional and non-emotional concepts according to the level of abstraction, the comparison framework is a dimension that goes from the abstract to the concrete, and it is being evaluated if the concepts of emotions are less abstract than the abstract concepts not referring to emotions. When analyzing the semantic content associated with the concepts, the frame of reference is no longer dimensional, but based on qualitative categories. These categories can be subtypes of concepts (Villani et al., 2019; Borghi, 2020), types of semantic content (Harpaintner et al., 2018) and other categories such as the cause-effect category used in this study. These different approaches are not mutually exclusive and can be used in combination.

The cause-effect attributes made it possible to clearly differentiate between emotional and non-emotional concepts, but not between concrete and abstract concepts. Also, the concepts of basic emotions generated more cause-effect attributes than those of secondary emotions. This could be explained by the role that concepts have in the social adaptation of people. Knowing why it occurs or what consequences an emotion generates does not have the same social importance as knowing if a tree produces oxygen. According to the theory of the conceptual act (Barrett, 2014), the concepts of emotion allow us to make sense of a set of affective and cognitive experiences that emerge in a given situation and also provide valuable information to predict our own behavior and that of others. As for the concepts of basic emotions, regardless of whether they refer to natural types (Ekman, 1999) or whether they are situated conceptualizations (Barrett, 2012), they differ from secondary emotions because they are experienced more frequently and are more salient, and this makes more emotional knowledge is relevant than facial expressions.

Regarding the attributes, the theory of the conceptual act (Barrett, 2014) grants an important role to emotional knowledge. This knowledge is essential when listing the different properties associated with a concept. The notable difference found between emotional and non-emotional concepts based on the cause-effect attributes corroborates this importance and at the same time allows us to maintain that there are qualitative differences between the emotional concepts and other types of concepts. However, the cause-and-effect schema could serve as a unifying element of the meaning of that experience. In addition, it could have a practical utility in social adaptation by allowing people to make predictions and decisions in everyday life.

The results show that emotion concepts are different from abstract concepts in some respects and similar in others. In this sense, the differences are not only quantitative but also qualitative if the cause-effect attributes are considered. The complexity of an emotional episode must be considered, not only because of the constellation of both internal and external experiences that constitute it, but also because they play a very important adaptive social role.

It is not yet clear whether emotions are discrete and culture-independent phenomena or whether they can be reduced to a few affective dimensions, however, both approaches have ample empirical evidence. Most likely, the answer lies somewhere in between. As the theory of the conceptual act proposes, there are basic biological phenomena that are at the base, not only of an emotional episode, but also of thoughts. An emotion is a multidimensional and very complex phenomenon that involves information that may or may not be conscious to the people who experience it, such as physiological changes. It has subjective components such as the feeling of emotion, the tendency to action, valence, activation, etc., and it has perceptual components external to people, such as facial expressions, vocals, posture, etc., it occurs in a context with social and psychological importance, and the way it is experienced can change according to the cultural knowledge acquired by the person. In this regard, it is more plausible that the concepts related to emotions, more than simply accounting for a natural category, constitute the center of gravity through which these different internal experiences, external perceptions and cultural knowledge come together and allow the realization of predictions that facilitate adaptation to the environment. In this framework, facial expressions may or may not emerge as a relevant factor, depending on the context and experimental design used.

Regarding the limitations, as has been pointed out, it should be considered that the selection of words for the experimental task and the difference in the number of concepts with a negative valence in basic emotions could have influenced the results, however, considering the study objectives, the selection of emotion concepts was determined by theoretical criteria (presence or absence of prototypical facial expressions) and the results obtained can be clearly interpreted from the underlying theories. Another aspect to consider is the interpretation of the results in a different cultural context. The concreteness averages of the words in this study are different (lower) than those reported by Pérez-Sánchez et al. (2021), which warns about the need to extend this normative study to the diversity of the Latin American context and to carry out comparative studies. However, our results are consistent with studies that propose that different subtypes of abstract concepts should be differentiated and that not only external perceptual aspects, but also internal ones and social interaction should be considered (Borghi, 2022). Finally, since this study had to be adapted for remote administration due to the COVID-19 pandemic, response latency could not be measured, and administration conditions could not be optimally controlled.

In conclusion, the results show that the presence of a prototypical perceptual referent could play a role in the perception of greater imageability and availability of context in the concepts of basic emotions with respect to the concepts of secondary emotions, but not in the perception of concreteness. This would be explained by the fact that the subjective feeling of emotion may have greater relevance in the rating of concreteness and facial expressions would have greater relevance in the rating of imaginability and availability of context. In addition, regarding the property lists, the cause-effect type attributes allowed to clearly differentiate the emotional concepts from the non-emotional ones, which would be explained by the relevance of the concepts of emotion to make predictions about one’s own behavior and the behavior of others in a social context. These results are consistent with the proposals of the need to differentiate between different types of abstract concepts and allow us to propose that emotional concepts are special, in that they differ from abstract concepts in both quantitative and qualitative aspects.

## Data availability statement

The raw data supporting the conclusions of this article are available on request from the authors.

## Ethics statement

The studies involving human participants were reviewed and approved by Comité Ético Científico de la Universidad de La Serena. The participants gave their informed consent to participate in this study.

## Author contributions

MG-A: preparation of the research project, project management, data analysis, discussion of results, and writing of the manuscript. DA: coordination of data collection and tabulation, search and systematization of references, and collaboration in writing the manuscript. Both authors contributed to the article and approved the submitted version.
